# Ex-Vivo Preservation with the Organ Care System in High Risk Heart Transplantation

**DOI:** 10.3390/life12020247

**Published:** 2022-02-07

**Authors:** Sebastian V. Rojas, Murat Avsar, Fabio Ius, David Schibilsky, Tim Kaufeld, Christoph Benk, Ilona Maeding, Michael Berchtold-Herz, Christoph Bara, Friedhelm Beyersdorf, Axel Haverich, Gregor Warnecke, Matthias Siepe

**Affiliations:** 1 Department of Cardiothoracic, Transplant and Vascular Surgery, Hannover Medical School, 30625 Hannover, Germany; avsar.murat@mh-hannover.de (M.A.); ius.fabio@mh-hannover.de (F.I.); kaufeld.tim@mh-hannover.de (T.K.); maeding.ilona@mh-hannover.de (I.M.); bara.christoph@mh-hannover.de (C.B.); Haverich.Axel@mh-hannover.de (A.H.); gregor.warnecke@med.uni-heildeberg.de (G.W.); 2 Department of Cardiovascular Surgery, University of Freiburg, 79106 Freiburg, Germany; david.schibilsky@universitaets-herzzentrum.de (D.S.); christoph.benk@universitaets-herzzentrum.de (C.B.); michael.berchtold-herz@universitaets-herzzentrum.de (M.B.-H.); Friedhelm.beyersdorf@universitaets-herzzentrum.de (F.B.); matthias.siepe@universitaets-herzzentrum.de (M.S.)

**Keywords:** heart transplantation, cardiac transplantation, ex vivo organ perfusion, organ care system, OCS heart

## Abstract

Objective: Ex vivo organ perfusion is an advanced preservation technique that allows graft assessment and extended ex situ intervals. We hypothesized that its properties might be especially beneficial for high-risk recipients and/or donors with extended criteria. Methods: We reviewed the outcomes of 119 consecutive heart transplant patients, which were divided into two groups: A (OCS) vs. B (conventional). Ex vivo organ perfusion was performed using the Organ Care System (OCS). Indications for OCS-usage were expected ischemic time of >4 h or >2 h plus given extended donor criteria. Results: Both groups included mostly redo cases (A: 89.7% vs. B: 78.4%; *p* = 0.121). Incidences of donors with previous cardiac arrest (%) (A: 32.4 vs. B: 22.2; *p* < 0.05) or LV-hypertrophy (%) (A: 19.1 vs. B: 8.3; *p* = 0.119) were also increased in Group A. Ex situ time (min) was significantly longer in Group A (A: 381 (74) vs. B: 228 (43); *p* < 0.05). Ventilation time (days) (A: 10.0 (19.9) vs. B: 24.3 (43.2); *p* = 0.057), postoperative need for ECLS (%) (A: 25.0 vs. B: 39.2; *p* = 0.112) and postoperative dialysis (chronic) (%) (A: 4.4 vs. B: 27.5; *p* < 0.001) were numerically better in the OCS group, without any difference in the occurrence of early graft rejection. The 30-d-survival (A: 92.4% vs. B: 90.2%; *p* = 0.745) and mid-term survival were statistically not different between both groups. Conclusions: OCS heart allowed safe transplantation of surgically complex recipients with excellent one-year outcomes, despite long preservation times and unfavourable donor characteristics. Furthermore, we observed trends towards decreased ventilation times and fewer ECLS treatments. In times of reduced organ availability and increasing recipient complexity, OCS heart is a valuable instrument that enables otherwise infeasible allocations and contributes to increase surgical safety.

## 1. Introduction

Fifty years after Barnard’s pioneering surgery, heart transplantation remains the gold standard therapy for terminal heart failure [1]. Transplantation has been improving quality of life and overall survival in critical patients [2]. However, limited donor organ availability and a growing number of heart failure patients are severe limitations. Currently, candidates for heart transplantation are significantly older, with higher levels of morbidity and more often bridged to transplant with mechanical circulatory support [3,4]. In addition, donor characteristics are changing towards older donors with an increasing incidence of risk factors such as diabetes or arterial hypertension, resulting in poorer organ quality, especially in Europe [5]. This aggravated the need of accepting marginal organs for heart transplantation [6]. Whereas important advances in recipient management, surgery and postoperative care (including immunosuppression) contributed to improve outcomes in heart transplantation, the technique for organ procurement and preservation has remained mainly unchanged: After administration of cold cardioplegia, hearts are retrieved and stored at 4 °C until the allograft is implanted in the recipient [7]. However, this approach has important disadvantages that can limit organ acceptance and viability: (1) Total ischemia time should not exceed 3–4 h, as the risk of early graft failure increases dramatically after this time frame. This is especially important because it not only limits the distance between donor and recipient hospital, but also involves accelerated surgery on the recipient side with the arriving donor heart [7]. Lately, this has become more relevant as a growing number of transplant candidates already undergone previous cardiac surgeries, including ventricular assist device (VAD) implantations [8,9]. In these patients, surgical dissection can be challenging and, due to tissue adhesions, time consuming [10]. Moreover, rushing the procedure to limit ischemic times increases the risk of bleeding [11]. (2) The standard preservation technique does not qualify for organ assessment. However, extended criteria organs should undergo further assessment in order to confirm viability. It is assumed that many potential organs are currently rejected because of the uncertainty of feasibility. However, a key strategy to counter the shortage of donor organs is to expand the organ pool by transplanting organs with extended criteria [12]. In this context, novel procurement techniques are required to warrant transplantation safety. The Organ Care System (OCS) (TransMedics, Inc., Boston, MA, USA) is designed to provide normothermic, ex vivo heart perfusion with oxygenated and nutrient supplemented donor blood [13,14,15]. It’s technology is based on Langendorff’s perfusion model, where perfusion of coronaries enables cardiac resuscitation and contraction.

The PROCEED II trial successfully demonstrated the non-inferiority of the OCS heart compared to the conventional procurement technique [16]. It was a multicentre, randomized study conducted in the US and Europe that showed equivalent outcomes regarding patient and graft survival between both study arms. However, significant differences in the OCS group included longer total preservation time and shorter cold ischemic time, demonstrating that the OCS heart enables longer out-of-the-body times in heart transplantation. Still, a major limitation of the PROCEED II trial is that it did not focus on outcomes of marginal organs or prolonged ischemic times. In contrast, in lung transplantation [17], the INSPIRE trial demonstrated a reduction of primary graft dysfunction in the OCS study arm [18,19]. In nations with the ability to DCD hearts, the use of OCS also allowed for the initiation of DCD heart transplantation programs [20,21,22,23,24,25].

From our local perspective, nationwide decreasing cases of heart transplantations, combined with increasing complexity of transplant candidates and poorer donor organ quality challenged us to find a solution capable of increasing number of transplantations but also warranting surgical safety for our recipients. Therefore, the aim of this study was to elucidate whether the OCS heart would be a safe alternative for high-risk heart transplantations.

## 2. Material and Methods

We performed a two-centre retrospective analysis of prospectively assessed registry data of all adult patients that underwent heart transplantation between January 2016 and January 2020 at our institutions. Patients were divided into two groups (Group A: OCS; n = 68 vs. Group B: Conventional; n = 51), according to organ preservation technique. High-risk cases were defined either by donor or recipient characteristics. Extended donor criteria included decreased LV function (LV-EF < 50%), LV hypertrophy (>13 mm), cardiopulmonary resuscitation, coronary artery disease, and expected prolonged ischemia time (>4 h). Recipient criteria that qualified for OCS procurement were: previous cardiac surgery, the presence of a ventricular assist device (LVAD, BIVAD) or pulmonary hypertension. The main objective of the present study was to assess the early outcome of heart transplantations using the OCS heart system for marginal donor organs or high-risk recipient/donor constellations. Events of interest included early mortality, incidence of adverse events like the necessity of postoperative VA-ECMO, postoperative dialysis, myocardial infarction, stroke, bleeding or pulmonary embolisms. The follow-up concluded one-year post transplantation.

**Figure 1:**
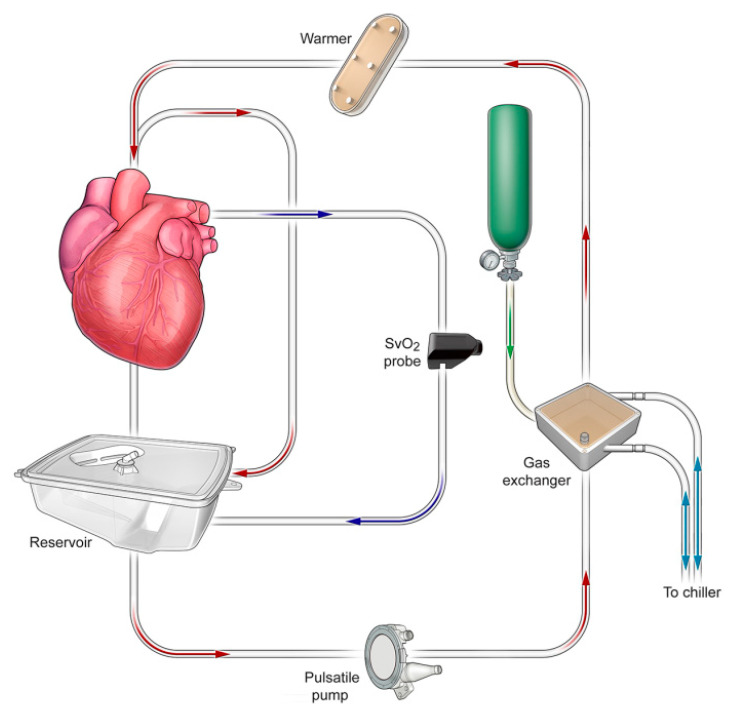
Schematic overview of the OCS HeartTM System and its components.

The OCS Heart system consists of a console including a wireless monitor and disposable heart perfusion modules that are required for every organ procurement (Figure 1). Hearts were instrumented and perfused in resting mode as stated previously [6,16]. The OCS requires approx. 1500 mL of donor blood, which is collected prior to organ retrieval. After the system is primed with donor blood, the retrieved heart is placed in the OCS heart module. Depending on individual characteristics (graft size, presence of ventricular hypertrophy or coronary artery disease) perfusion is initiated, usually with an aortic flow of 1 L/min. Warm blood, substituted with epinephrine, nutrients and a specific maintenance solution is pumped into the ascending aorta, filling both coronaries and feeding the myocardium. After ventricular perfusion, venous blood drainage through the coronary sinus culminates in blood accumulation in the right atrium. As both cava veins are temporary occluded during OCS perfusion, blood is then ejected through the right ventricle into the pulmonary artery, where a cannula is placed that returns the deoxygenated blood to the perfusion circuit and enables the measuring of coronary flow, which is a central parameter of control in OCS heart perfusion [7]. Assessment of arterial and venous blood samples enables the monitoring of lactate levels which are markers for adequate organ perfusion. Thus, adjustments of aortic (pump) flow and target aortic pressure can be performed during OCS heart preservation.

### 2.1. Exclusion Criteria

OCS exclusion criteria for donor hearts previous to organ procurement were: Donor haematocrit <25% without possible blood transfusion, continuous norepinephrine or adrenaline application >0.5 μg/kg/mL and severe cardiac contusion. In addition, two marginal organs were declined in the OCS due to rapidly increasing venous and arterial lactates (>5 mmol) at initiation of OCS perfusion. Recipients were intubated as soon as hearts were accepted in the OCS. Heart transplantation was performed as previously described [10].

Ethical approval for retrospective data analysis was obtained (137/11, University of Freiburg, Freiburg, Germany) and patient consent was waived.

### 2.2. Statistical Analysis

Statistical analysis was performed using SPSS 24.0 (IBM SPSS Statistics, IBM Corp., Armonyk, NY, USA). Most continuous variables were normally distributed and were summarized as median M ± interquartile range [IQR] or standard deviation (SD). We utilized Fisher’s exact test (baseline, donor characteristics, early outcome), the Wilcoxon-Mann-Whitney-Test (baseline, donor characteristics), the unpaired t-test (baseline, donor characteristics, intraoperative data), Pearson’s Chi-Square test (baseline characteristics, donor characteristics), and the Kaplan–Meier survival estimation for statistical analysis depending on the scale level, the distribution and the number of groups. Survival curves were compared applying the log rank test. Differences were considered significant at *p* < 0.05.

## 3. Results

Recipient baseline characteristics are summarized in Table 1 and were similar in both groups. Most recipients were male with mean ages of 49.4 (12.6) (group A) and 58.6 (13.1) years (group B) (*p* = 0.721). The majority of patients fulfilled “high urgency” (HU) criteria for heart transplantation, according to Eurotransplant (ET) classification (Group A: 86.8% vs. Group B: 86.3%; *p* = 1.0), with a mean waiting time of: Group A: 670 (1039) d and Group B: 688 (920) (*p* = 0.468), respectively. A total of 61 patients (89.7%) in the OCS cohort underwent previous cardiac surgery compared to 40 (78.4%) in the control group (*p* = 0.121). The presence of a left ventricular assist device (LVAD) was found in Group A (n = 53; 77.9%) and Group B (n = 38; 74.5%) patients (*p* = 0.67). Moreover, 20.8% (Group A) and 27.0% (Group B) of patients also underwent previous VAD exchange surgery.

Donor characteristics are summarized in Table 2. Donor age in Group A was 42.7 (12.3) years, compared to 40.1 (14.2) years in group B (*p* = 0.45). A total of 13 group A donors (19.1%) showed LV hypertrophy compared to four (8.3%) Group B donors (*p* = 0.119). Mean ejection fraction of group A donor hearts was 61.3 (5.7)% and 60.3 (5.1)% in group B donors. The incidence of previous cardiac arrest in donors was 32.4% in Group A and 22.2% in Group B donors (*p* = 0.029). Accordingly, mean cardiac arrest time was 22.4 (14.2) min and 14.6 (7.3) min for OCS and non-OCS donors, respectively (*p* = 0.155). The most important cause of death for donors was cerebral haemorrhage (Group A: 47.9 vs. Group B: 47.1%). The most common gender matches were male-male (Group A: 60.3% vs. Group B: 54.9%) and female-female (Group A: 20.6 vs. Group B: 17.6%) in both groups.

**Figure 2:**
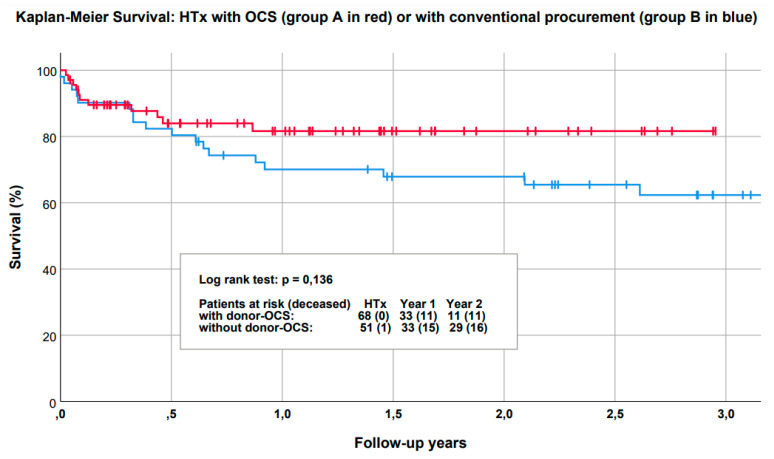
Comparison of survival showed no significant difference between the OCS heart group and controls. The red line represents survival of the OCS arm, whereas the blue line represents the survival curve of transplant patients that received an organ procured by the conventional method.

Perioperative data is demonstrated in Table 3. Mean total ischemia time in Group B was 228 (43) min. In contrast, Group A showed lower mean ischemic times of 115 (43) min, with a mean out-of-the-body-time of 381.0 (74.0) min. Accordingly, the mean OCS run time was 267.0 (54.3) min, not including pre- and post-OCS ischemia times (pre OCS ischemia: 42.4 (15.8) min; post OCS ischemia: 76.6 (33.6) min). The longest OCS perfusion time was 438 min. Detailed early outcome is described in Table 4, whereas comparison of survival between both groups is plotted in Figure 2. There were no statistically significant differences regarding patient survival in both groups (*p* = 0.136). Mean ventilation time was 10.0 (19.9) days in Group A and 24.3 (43.2) days in Group B patients (*p* = 0.057). Mean ICU stay was in Group A: 25.1 (39.0) days compared to 24.6 (46.9) days in Group B *(p* = 0.526). The incidence of postoperative VA-ECMO treatment was present in both groups (Group A: 25.0% vs. Group B: 39.2%; *p* = 0.112), with almost equal mean duration times (Group A: 10.1 (7.8) d vs. Group B: 7.3 (5.3) d). Permanent dialysis was increased in the conventional group (Group B: 27.5% vs. Group A: 4.4%; *p* < 0.001). There were no cases of myocardial infarction. Incidence of rejection (>R1 ISHTL classification) was 23.5% in Group A and 25.5% in Group B patients (*p* = 0.83). One-year survival was 89% in Group A patients, compared to 85% in Group B (*p* = 0.225) (Figure 2). A total of two hearts were investigated in the OCS but not used for transplantation due to exclusion criteria defined in Material and Methods.

## 4. Discussion

The best treatment option for end stage congestive heart failure is heart transplantation [26]. However, the effectiveness of this treatment has been hampered by a growing mismatch between patients on the waiting list and available donor organs. Thus, an increasing number of patients are now frequently bridged to transplantation with left ventricular assist devices (LVAD) [26]. Despite the fact that LVAD therapy has dramatically improved during the past two decades, there are still many VAD-associated complications like stroke, aortic valve insufficiency, right heart failure, pump thrombosis, gastrointestinal bleeding or infection that make urgent transplantation necessary and are common indications for priority on the waiting list [27,28,29]. Thus, LVAD therapy can be considered both blessing and curse, on one hand saving patients from imminent death due to a failing heart, but also generating complex transplant candidates with increased surgical risk profiles and ongoing complications, capable of jeopardize transplant outcomes. This is reinforced by the fact that only LVAD related complications qualify for high-urgent transplantations according to the current allocation algorithms of Eurotransplant. Even if there is an ongoing controversy about the questions as to whether there is an increased operative risk in bridge-to-transplant-VAD candidates, from our perspective, it is undisputable that a transplant candidate with ongoing LVAD complications will have a higher surgical risk profile for heart transplantation than patients that had not been operated on previously. Moreover, the latest demographical developments aggravated this involuntary marriage between heart transplantation and LVAD therapy, making bridge-to-transplant therapy indispensable in modern heart failure treatment [26,30]. In our opinion, this clearly changed the transplant scenery, moving from decreasing transplant numbers in the first years of LVAD therapy with decreasing outcomes, to improvements in the past years where surgeons have adopted novel strategies in heart transplantation. Whereas immunosuppression and overall patient management have markedly improved during past decades in heart transplantation, donor management and procurement techniques have not. During the past 50 years, the vast majority of hearts have been preserved by applying cold storage. This technique is safe, but confines transplantation to ischemic times below 4 h, limiting the distance between donor hospitals and transplant centres. On the other hand, donors have become not only older but also scarce, and there is a clear trend towards accepting donor hearts with extended criteria [6,31]. However, for these cases, further physiological evaluation of the donor organs becomes necessary in order to warrant transplantation safety for the recipients. Ex vivo heart perfusion has been effective to preserve donor hearts for transplantation guided by lactate profiling during perfusion [7]. In their seminal study, García Sáez et al. presented a single centre, single-arm experience of successfully using the OCS heart in transplantations with adverse donor/recipient profiles [6]. Since the situation of heart transplantation has changed towards more complex procedures with marginal donor organs, we decided to incorporate this novel technique (OCS Heart, Transmedics, Andover, MA, USA) with the aim of improving the safety and quality of cardiac transplantation in cases where either donor characteristics, recipient physiognomies, or both made the transplantation a high risk one. In the present study we retrospectively analysed a total of 119 adult patients that underwent cardiac transplantation in our centres. Despite the fact that we did not perform propensity matching, there were no significant differences between both groups in the majority of baseline characteristics (Table 1). In both groups, a high patient percentage showed previous surgical interventions, including long-term mechanical circulatory support (i.e., LVAD). The fact that almost one quarter of all recipients already underwent VAD exchange before transplantation emphasizes the long waiting times on the transplant list. Despite the fact that most of our recipients were categorized as “high urgency” (HU according to Eurotransplant) candidates, the mean waiting time was above one year. Regarding donor characteristics, Group A donors (OCS) were slightly older with a higher presence of ventricular hypertrophy, a higher incidence of previous cardiac arrest, and prolonged resuscitation intervals. All donations were after brainstem death, (DBD) and approximately half of them were due to cerebral haemorrhage, which is representative for our current regional situation. Mean out-of-the-body times in group A were by far longer than the total ischemia times in the control group (6.4 h vs. 3.8 h). There was also a tendency towards longer operation times in the OCS group, which is possibly related to the more complex donor and recipient management but did not have impact on the overall outcome. We observed an 89% one-year-survival in the OCS group, which was comparable to Group B. In addition, patients in Group A showed lower ventilation times (*p* = 0.057), a lower incidence of postoperative ECMO treatment (*p* = 0.112), and shorter overall hospital stays (*p* = 0.43). The incidence of acute kidney failure requiring dialysis was high in group B (70.6%) and lower in the OCS group (52.9%, *p* = 0.06). Moreover, at discharge, only 4.4% of Group A patients still required dialysis (*p* < 0.001). The incidences of other adverse events like acute rejection and MACE were comparable to available register data and did not differ among them. 

We consider that a major strength of the study is that it describes the real-world experience of the OCS heart outside of a clinical trial. It summarizes the practice of two major independent transplant centres with good operative outcomes despite complex donor and recipient configurations. With the obtained routine of ex vivo heart perfusion and the earned knowledge, further clinical studies in the field can be developed for further evaluation.

The retrospective nature of the study and missing randomization are its main limitations. We decided not to perform a propensity score matching since baseline characteristics were similar in both groups. Further limitations of the study include the selection bias, since the inclusion criteria allow for some subjectivity (“expected ischemic time”). Concerning the selection bias, it is very clear that more risky combinations of donor and recipient profiles were included in the OCS group, since in both centres some transplantations were only deemed possible with the use of OCS. This extended donor criteria selection bias was only partly reflected by the data. The fact that OCS use was not paid for by the insurance companies but had to be financed by the hospitals underscores that OCS use was certainly not done in low risk and easily manageable circumstances which had best chances with conventional procurement. We consider that the main strength of our study.

## 5. Conclusions

Changing environments in cardiac transplantation is encouraging heart failure practitioners to find novel strategies that contribute to increase the safety and efficacy of heart transplantation in the context of increased surgical complexity. Not only were most of our patients bridged to transplantation with assist devices, making the surgery itself more difficult in relation to ischemia time, but the donors have also changed given the extended criteria that are now used. Therefore, the OCS heart is a promising method that enables the procurement of donor hearts in more distant hospitals and also gives space to careful surgical preparation. It also allows, through the evaluation of hearts with extended criteria, for the to augmentation of the donor organ pool. Even if our study underlies the obvious limitations of retrospective analysis, we experienced excellent surgical and mid-term survival rates. Therefore, we would recommend the use of ex vivo heart perfusion in cases of heart transplantation with increased complexity.

## Figures and Tables

**Table 1:** Recipient baseline characteristics.

	Group A (OCS)	Group B (Non OCS)	*p*
Age (y) M (SD)	49.4 (12.6)	58.6 (13.1)	0.72
Gender (male) (%)	76.5	78.4	0.83
Weight (kg) M (SD)	81.0 (13.8)	81.5 (17.1)	0.85
Height (cm) M (SD)	177 (8)	176 (10)	0.85
BMI M (SD)	26.0 (4.2)	26.1 (4.4)	0.84
Cardiac index M (SD)	2.3 (0.6)	2.4 (0.7)	0.68
PVR (dyn/s/cm^2^) M (SD)	142 (60)	146 (92)	0.83
HU grade (%)	86.8	87.7	1.00
Waiting-list period (days) M [IQR]	137 [607]	239 [903]	0.47
Dialysis pre HTx (%)	5.9	6.0	0.83
Stroke pre HTx (%)	14.7	22.0	0.34
Previous cardiac surgery (%)	89.7	78.4	0.67
Presence of durable VAD (%)	77.9	74.5	0.67
Previous VAD exchange (%)	20.8	27.0	0.61
Heart failure etiology			
ICM n (%)	22.1	35.3	*
DCM n (%)	57.4	43.1	*
Congenital heart disease (%)	4.4	3.9	*
Other (%)	4.4	5.9	*

* Pearson’s Chi^2^-Test (DCM vs. ICM vs. congenital vs. other): *p* = 0.385; Fisher’s-Exact-Test (DCM vs. ICM): *p* = 0.125. Abbreviations: BMI: Body Mass Index, PVR: Pulmonary Vasculary Resistance, HU: High Urgency, HTx: Heart Transplantation, VAD: Ventricular Assist Device, ICM: Ischemic Cardiomyopathy, DCM: Dilative Cardiomyopathy.

**Table 2:** Donor characteristics.

	Group A (OCS)	Group B (Non OCS)	*p*
Donor age (y) M [IQR]	37 [25]	44.5 [16]	0.45
Donor height (cm) M (SD)	176 (9.0)	178 (9.3)	0.30
Donor weight (kg) M (SD)	81.0 (14.0)	80.8 (15.3)	0.93
Donor BMI M (SD)	26.2 (3.5)	25.5 (4.1)	0.39
Donor LV-EF (%) M [IQR]	60 [5]	60 [3]	0.38
Donor gender (male) (%)	63.2	58.8	0.71
Donor LV-hypertrophy (%)	19.1	8.3	0.119
Donor coronary sclerosis (%)	2.9	2.2	0.119
Donor cardiac arrest (%)	32.4	22.2	0.029
Donor cardiac arrest period (min) M (SD)	22.4 (14.2)	14.6 (7.3)	0.155
CAUSE OF DEATH			
Cerebral hemorrhage (%)	48.5	47.1	*
Cerebral ischemia (%)	23.5	17.6	*
Head Trauma (%)	7.4	25.5	*
Other (%)	20.6	9.8	*

* Pearson’s Chi^2^-Test (cerebral hemorrhage vs. cerebral ischemia vs. head trauma vs. other): *p* = 0.385.

**Table 3:** Perioperative Heart Transplantation Data.

	Group A	Group B	*p*
ISCHEMIA			
Pre OCS ischemia (min) M (SD)	42.4 (15.8)		
OCS running-time (min) M (SD)	267 (54.3)		
Post OCS ischemia (min) M (SD)	76.6 (33.6)		
Pre & Post OCS ischemia (min) M (SD)	115 (43.1)		
OCS Ex-situ-time (min) M (SD)	381 (74.0)		
Ischemia non-OCS patients (min) M (SD)		228 (43)	
SURGERY			
Operation time (min) M (SD)	489 (94)	458 (131)	0.165
GENDER-MATCH			
Recipient:donor = male:male (%)	60.3	54.9	
Recipient:donor = female:female (%)	20.6	17.6	
Recipient:donor = male:female (%)	16.2	23.5	
Recipient:donor = female:male (%)	2.9	3.9	

**Table 4:** Early outcome.

	Group A	Group B	*p*
Ventilation time (d) M [IQR]	22 [37.8]	47.4 [105]	0.057
ECMO postop. n (%)	25.0	39.2	0.112
Duration ECMO (d) M [IQR]	6.8 [7.3]	6.7 [5.8]	0.27
ICU stay (d) M [IQR]	36 [48]	70 [118]	0.53
Total postoperative stay (d) M [IQR]	96 [71]	111 [106]	0.49
Bleeding requiring surgery (%)	19.1	25.5	0.50
Dialysis (acute) (%)	52.9	70.6	0.060
Dialysis (permanent) (%)	4.4	27.5	0.001
Myocardial infarction (%)	0	0	-
Pulmonary embolism (%)	1.5	3.9	1.00
Stroke (%)	4.4	5.6	1.00
Rejection grade (>1R) (%)	23.5	25.5	0.83

## Data Availability

Data can be obtained upon request to the authors.

## Abbreviations

BIVAD	Biventricular assist device
DBD	Donation after brainstem death
DCD	Donation after cardiac death
ET	Eurotransplant
ECMO	Extracorporeal membrane oxygenation (VA = veno-arterial)
HU	High urgency
ICU	Intensive care unit
LV	Left ventricle
LVAD	Left ventricular assist device
LV-EF	Left ventricular ejection fraction
MACE	Major adverse cerebrovascular event
OCS	The Organ Care System
VAD	Ventricular assist device
